# Homozygous TBC1 domain-containing kinase (TBCK) mutation causes a novel lysosomal storage disease – a new type of neuronal ceroid lipofuscinosis (CLN15)?

**DOI:** 10.1186/s40478-018-0646-6

**Published:** 2018-12-27

**Authors:** Stefanie Beck-Wödl, Klaus Harzer, Marc Sturm, Rebecca Buchert, Olaf Rieß, Hans-Dieter Mennel, Elisabeth Latta, Axel Pagenstecher, Ursula Keber

**Affiliations:** 1 Department of Medical Genetics and Applied Genomics, University of Tübingen, Tübingen, Germany; 2 Department of Neuropediatrics and Neurometabolic Laboratory, Children’s Hospital of the University of Tübingen, Tübingen, Germany; 3 Department of Pediatrics, University Hospital of Marburg, Marburg, Germany; 4 Department of Neuropathology, Philipps University and University Hospital of Marburg, Baldingerstrasse, 35043 Marburg, Germany

**Keywords:** Infantile muscular hypotonia with psychomotor retardation and characteristic facies 3 (IHPRF3), Central nervous system (CNS), Vacuolated lymphocytes, Autophagy, Mammalian target of rapamycin (mTOR), Rab

## Abstract

Homozygous mutation of TBC1 domain-containing kinase (*TBCK*) is the cause of a very recently defined severe childhood disorder, which is characterized by severe hypotonia, global developmental delay, intellectual disability, epilepsy, characteristic facies and premature death. The link between TBCK loss of function and symptoms in patients with TBCK deficiency disorder (TBCK-DD) remains elusive. Here we demonstrate for the first time the histopathological characteristics of TBCK deficiency consisting of 1) a widespread and massive accumulation of lipofuscin storage material in neurons of the central nervous system without notable neuronal degeneration, 2) storage deposits in few astrocytes, 3) carbohydrate-rich deposits in brain, spleen and liver and 4) vacuolated lymphocytes. Biochemical examinations ruled out more than 20 known lysosomal storage diseases. These investigations strikingly uncover TBCK-DD as a novel type of lysosomal storage disease which is characterized by different storage products rather than one specific type of accumulated material. Due to the clear predominance of intraneuronal lipofuscin storage material and the characteristic clinical presentation we propose to classify this disease as a new subtype of neuronal ceroid lipofuscinosis (CLN15). Our results and previous reports suggest an autophagosomal-lysosomal dysfunction caused by enhanced mTORC1-mediated autophagosome formation and reduced Rab-mediated autophagosome-lysosome fusion, thus disclosing potential novel targets for therapeutic approaches in TBCK-DD.

**Electronic supplementary material:**

The online version of this article (10.1186/s40478-018-0646-6) contains supplementary material, which is available to authorized users.

## Introduction

Homozygous mutation of TBC1 domain-containing kinase (*TBCK*) leads to a very recently defined severe disorder in childhood, which is characterized by infantile muscular hypotonia, psychomotor retardation and characteristic facies (IHPRF3; OMIM: 616900). To date, more than 30 patients with various homozygous *TBCK* mutations have been reported [1, 4, 9, 17, 20, 31, 35, 51]. The disease is often accompanied by global developmental delay, distinctive facial features like deeply set eyes and tented upper lip vermilion, medication refractory epilepsy and chronic respiratory failure [51]. Typical brain imaging signs are brain atrophy and progressive leukoencephalopathy with a thinned corpus callosum. The disease has a generally short survival and only exceptional clinical courses up to two decades have been described [17, 35].

In all reported cases, the different *TBCK* mutations resulted in aberrant TBCK protein. The knowledge about the function of TBCK is still limited. The protein contains a Tre-2/Bub2/Cdc16 (TBC) domain, a rhodanase-like domain and a kinase domain, which has been proposed to be inactive due to a lack of essential catalytic subdomains [7, 29, 42]. The TBCK protein is expressed in most organs (https://www.proteinatlas.org/). It has been shown to suppress cell proliferation [29, 50] and to play a role in cell growth and actin organization by enhancing the signalling pathways of mammalian target of rapamycin (mTOR), presumably at a transcriptional or post-transcriptional level [29]. Interestingly, an autophagosomal-lysosomal dysfunction has been described recently in patients with TBCK deficiency [35] that is attributed to the disturbed activation of the mTOR complex 1 (mTORC1), which regulates autophagy [6]. In addition, it has been suggested that *TBCK* encodes a Rab GTPase-activating protein [9].

Despite these data, the actual mechanisms linking *TBCK* gene mutation to the clinical phenotype remain elusive, thus impeding the establishment of potential therapeutic strategies. This study provides the first autopsy reports of two siblings, who suffered from homozygous *TBCK* mutation. Macroscopic, histological and ultrastructural investigations give insights into the cellular changes in the disorder and provide compelling evidence for classification of TBCK deficiency disorder (TBCK-DD) as a novel type of lysosomal storage disease (LSD).

## Materials and methods

### General study design

Two siblings born in 1972 and 1974 suffered from the same severe and at that time unknown disease. Clinical examinations of both patients rendered no definite diagnosis. Autopsies were done immediately after death of patient 1 in 1978 and of patient 2 in 1985. Investigations included macroscopic, histological and biochemical analysis, but no definite diagnosis could be made. With the recent advent of modern genetic techniques it became possible to pinpoint the cause of the disorder. Subsequently, intense re-evaluation of tissue samples including completive immunohistochemical and ultrastructural studies was performed. Written informed consent to participate in the study and for publication of the clinical photographs (Fig. 2) was obtained from the parents of the siblings.

### Molecular gene analysis

Genomic DNA was isolated from spleen tissue of patient 2 using the QIAamp Mini Kit (Qiagen NV, Hilden, Germany) following the manufacturer’s instructions. Mutations in the *TBCK* gene were uncovered by whole exome sequencing. Target regions were enriched using the SureSelectXT Human All Exon Kit V5 (Agilent, Böblingen, Germany) according to the manufacturer’s protocol. Sequencing was performed on a HiSeq2500 instrument (Illumina, San Diego, CA; USA). On average 100 million paired-end reads with a length of 125 bp were produced per exome. The conversion of the sequence data in the FASTA format was done by Illumina bcl2fastq. Adapter sequences were removed with SeqPurge (https://github.com/imgag/ngs-bits) and the trimmed reads were mapped to the human reference genome hg19 (GRCh37) using Burrows Wheeler Aligner (http://bio-bwa.sourceforge.net). PCR-duplicates were removed with samblaster (https://github.com/GregoryFaust/samblaster). Deletions and insertions were realigned with ABRA (https://github.com/mozack/abra). Variants were detected using freebayes (https://github.com/ekg/freebayes) and transcript/protein information was annotated with SnpEff / SnpSift (http://snpeff.sourceforge.net).

Filtering of variants for pathogenicity was performed with an in-house tool (MS, unpublished). Calls with an allele frequency ≥ 1% in the 1000 Genomes, ExAC or Kaviar databases were excluded. In addition, frequently observed variants in our in-house database (≥ 20x) were removed. All exonic non-synonymous variants including splice sites which potentially change the protein were taken into account (other intronic and UTR mutations were eliminated). Finally the remaining SNVs and INDELs of the index patient were checked for the three modes of inheritance: Autosomal dominant (de novo?), autosomal recessive, X-linked recessive or dominant and the coverage (minimum 20x).

Sanger sequencing of the *TBCK* gene was performed on genomic DNA from spleen and paraffin-embedded brain tissue of patient 2. Genomic DNA was also isolated from peripheral blood leukocytes of both parents by standard methods in order to confirm the pathogenic variants and exclude de novo mutations. The following primer sequences were used: *TBCK*-Ex3F: AGCCCTTTCGTGGAAGAACC, *TBCK*-Ex3R: GCCCTGATCCCAGTTGCT, chr4: 107183639–107183188. *TBCK* reference sequence was NM_001163435.2, ENST00000394708.6 and all genomic positions are denoted according to GRCh37/hg19.

### Biochemical analysis

Enzymatic, cytochemical and thin-layer chromatographic lipid analyses screening more than 20 LSDs were performed on white blood cells, cultured fibroblasts and frozen tissue samples of brain, spinal cord and liver obtained from autopsy of both patients. Additionally, urine of patient 2 was tested for oligosaccharide concentration and spleen tissue of patient 2 was examined regarding its content of carbohydrate positive (CHp) material. For this, buffer extract from 200 mg spleen tissue was delipidated by phase partitioning with chloroform/methanol 2:1 by volumes. The aqueous upper phase was dialyzed against water, concentrated and applied to a BioGel P-4 column [25]. The buffer-eluted fractions were monitored for CHp material by spotting aliquots to a thin-layer plate and reacting the spots with anisaldehyde/sulfuric acid reagent (characteristic colour for carbohydrates, glucose as a reference). CHp material was detected only in some fractions eluted with the void volume. These fractions were also positive with a mucopolysaccharide reagent [13]. Positive fractions were pooled and centrifuged. The resulting sediment and supernatant were again tested for CHp material as follows: The test spots were applied to a start line 1 cm above the lower edge of a thin-layer plate. Water-soluble, low-molecular-weight contaminations were removed from the start line by running the plate upwards with a polar solvent system for 2 h. Finally, the run plate was sprayed with anisaldehyde reagent to visualize CHp material. The entire procedure was also applied to spleen tissue from two control individuals.

### Histological and immunohistochemical analysis

Autopsies were done immediately after death of patient 1 in 1978 and of patient 2 in 1985. Visceral and central nervous tissue was formalin fixed and paraffin embedded according to standard protocols. Multiple stained sections of all relevant brain regions and of the spinal cord were available from patient 1 and re-evaluated for this study. From patient 2, both the stained sections and archived paraffin blocks of the central nervous system and peripheral organs were re-evaluated and used for new histological and immunohistochemical stains, respectively. Hematoxylin and eosin, Klüver Barrera (luxol fast blue and cresyl violet), Sudan black and red, periodic acid-Schiff (PAS), Alcian blue, Shimizu and Heidenhain-Woelcke stains as well as Gallyas silver impregnation were performed according to standard procedures. Investigation of sections was performed with normal or differential interference contrast microscopy and autofluorescence was evaluated with light excitation using ultraviolet (excitation wavelength 340–380 nm), blue (460–500 nm) and green (515–560 nm) light.

For immunohistochemistry, heat-induced epitope retrieval was performed either with citrate or EDTA according to the manufacturer’s protocol of the respective primary antibody. Sections were incubated for one hour with the following primary antibodies: rabbit anti-GFAP (1:1000; Dako Z0334), mouse anti-ß-Amyloid (1:100; Dako M0872), rabbit anti-p62 (1:100; Enzo BML-PW9860), mouse anti-CD3 (1:50; Novocastra Laboratories NCL-CD3-PS1), mouse anti CD20 (1:400; Dako M0755), mouse anti-CD68 (1:100; Dako M0876) and mouse anti-CD138 (1:200; Dako M7228). Sections were washed and incubated with post-block solution and HRP-polymer reagent according to the manufacturer’s protocol of ZytoChem-Plus HRP Polymer-Kit (Zytomed Systems).

### Ultrastructural analysis

For ultrastructural analysis, cylinders of 3 mm in diameter were punched out of paraffin embedded tissue of the neocortex, medulla oblongata and spinal cord anterior horn, respectively. We chose areas in which neuronal storage inclusions were histologically observed. The tissue was rehydrated and fixed in glutaraldehyde. Tissue preparation was performed as described previously [26]. In brief, the tissue cylinders were fixed in buffered glutaraldehyde, postfixed in osmiumtetroxide and embedded in Epon resin. Thin sections were contrasted with uranylacetate and lead citrate and analyzed using a Zeiss EM 902.

## Results

### Clinical report

**Fig. 1:**
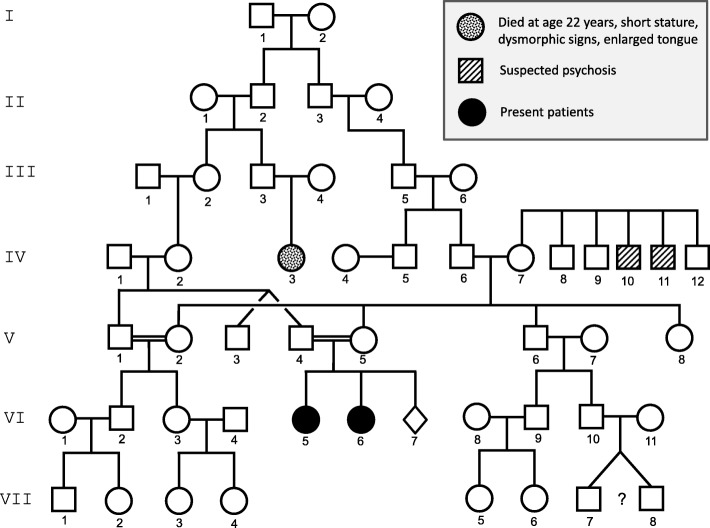
Family tree of the patients. The siblings (VI.5 and VI.6, black circles) were born to Caucasian, distantly consanguineous parents, who did not suffer from the disease. ◊, pregnancy with induced abortion. ?, twins with unknown zygosity

The two sisters were born to healthy Caucasian German parents with distant consanguinity (Fig. 1). The third pregnancy was interrupted without a prenatal diagnosis. The clinical symptoms, age of onset and age of death of both patients are summarized in Table 1.

**Table 1:** Clinic of siblings

	Patient 1	Patient 2
Age of onset	4 months	10 months
Age of death	7 years	11 years
Cause of death		
Respiratory failure	+	+
Main symptons		
Severe Hypotonia	+	+
Global developmental delay	+	+
Intellectual disability	+	+
Generalized onset seizures	+	+
Reduced visual acuity	–	+
Blindness	+	–
Appearance		
Undersize	+	+
Underweight	–	+
Short neck	+	+
Facial dysmorphia	+	+
Open mouth	+	+
Tented upper lip	+	+
Macroglossia	+	+
Furrowed tongue	+	+
Unilateral esotropia	+	+

**Fig. 2:**
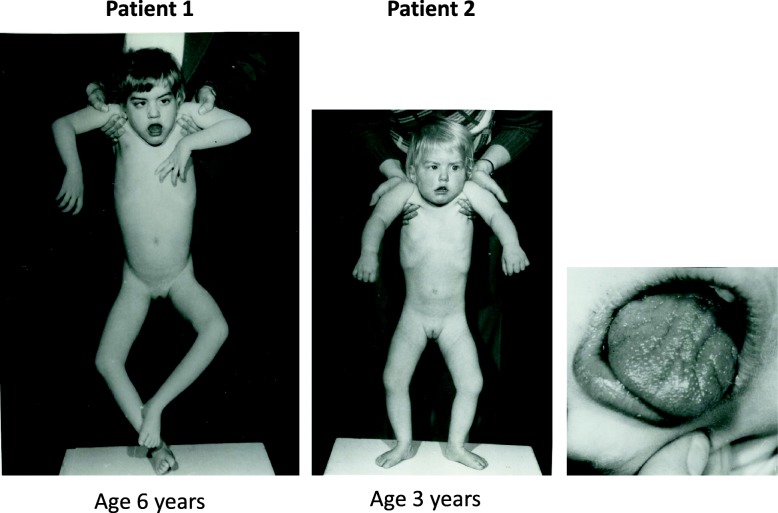
External appearance of the two patients. Severe hypotonia, a short neck and mild facial dysmorphia with open mouth, tented upper lip vermilion, macroglossia, furrowed tongues and right esotropia are seen

In detail, patient 1 started to suffer from hypotonia and loss of tendon reflexes at the age of about 4 months. The progressive hypotonia prevented any statomotor development except for lifting the head in prone position, an ability that was lost later on. At the age of 7, the body size was reduced (1.2 m, according to the 30. percentile of age-related WHO reference values), the body weight was regular (31.5 kg). X-ray examination revealed no signs of dysostosis. She had a short neck and mild facial dysmorphia with an open mouth, tented upper lip vermilion, macroglossia, furrowed tongue and right esotropia (Fig. 2). She was intellectually disabled, never able to speak and blind, but able to hear. At the age of 5 years, epilepsy with generalized onset motor seizures became manifest. Accordingly, electroencephalographic (EEG) waves of severely changed general activity and some hypersynchronous activity were observed. The patient died at the age of 7 years and 3 months from a bronchopneumonia with respiratory failure.

Patient 2, the younger sister, developed similar clinical features of a profound global developmental delay with a slightly later onset and longer survival. Her statomotor maximum was the all-fours position, when the symptoms with hypotonia started at the age of 10 months. The subsequent disease progress was faster compared to her sister and generalized onset motor seizures appeared soon. She was intellectually disabled, never able to speak and suffered from severely reduced visual acuity. Hearing was intact and she developed hyperacusis. The external appearance was comparable to her sister (Fig. 2, middle and right panel). Size and weight was normal at birth but severely reduced by the age of 11 years (size 1.24 m, according to < 1. percentile and weight 33 kg, according to the 22. percentile of age-related WHO reference values, respectively). Electroneurographic and myographic measurements at the age of 9 years revealed reduced distal nerve conduction velocities and a complete absence of spontaneous and arbitrary muscular activity. Computer tomography showed a general cortical, frontally accentuated atrophy with slightly distended, deformed ventricles and a massive atrophy of the lower cerebellar vermis. The patient died at the age of 11 years from bronchopneumonia with respiratory failure.

### Genetic investigations

**Fig. 3:**
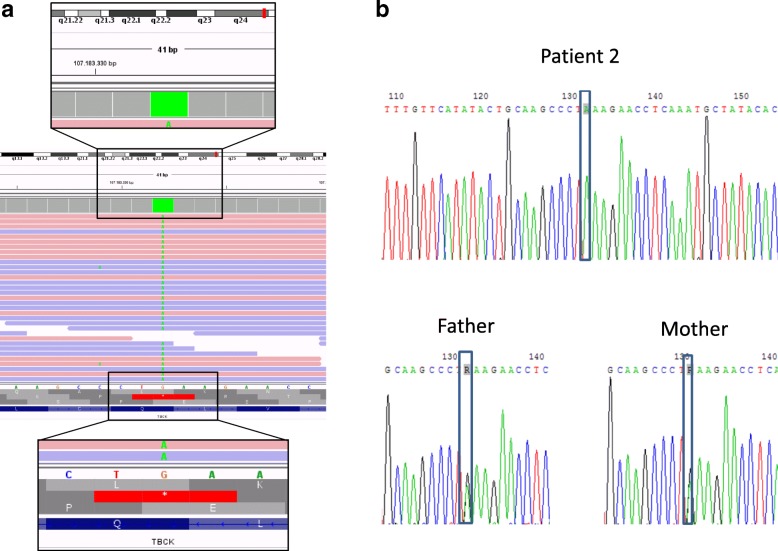
The TBCK defect in the genome of patient 2 and her parents. **a**, Integrative Genomics Viewer presentation of the homozygous stop mutation in the *TBCK* gene of patient 2. Below the green square, green dots show the base exchange (c.304C > T) in the multiple reads. **b**, Sanger sequencing of the *TBCK* gene of patient 2 and her parents. Sequencing was performed on the reverse strand, showing the base exchange G > A in our patient (grey boxes). Both parents carry the same mutation heterozygously, as seen in the R (grey boxes) that resembles an A and a G in each allele

Whole exome sequencing of the DNA of patient 2 yielded 77 million mapped reads with a mean coverage of more than 94%. The analysis revealed a homozygous nonsense mutation in exon 3 of *TBCK*: NM_001163435.2:c.304C > T, p.Gln102* (Fig. 3a). This leads to a premature stop codon and affects the protein kinase domain. The mutation was confirmed by Sanger sequencing and accordingly heterogeneously present in both parents of the patients (Fig. 3b). Other gene mutations, especially of known metabolic diseases, were not detected.

### Biochemical investigations

Biochemical analyses were performed on white blood cells, cultured fibroblasts and frozen tissue samples of patient 2 and ruled out the following metabolic diseases: metachromatic leukodystrophy, multiple sulfatase deficiency, Krabbe disease, GM1 gangliosidosis I and II, GM2 gangliosidosis, galactosialidosis, sialidosis, Salla disease, Fabry disease, Schindler disease, Farber disease, Niemann-Pick disease, Gaucher disease, mucopolysaccharidoses (7 types), Wolman disease, cholesterylester storage disease and neuronal ceroid-lipofuscinosis (NCL). Thin-layer chromatographic lipid analyses of brain, spinal cord and liver tissues showed normal patterns of gangliosides including GM_1_, GM_2_ and GM_3_, and of glucosylceramide, galactosylceramide, sulfatides, and sphingomyelins. Urinary oligo- and mucopolysaccharides were unremarkable.

Investigation of carbohydrate-positive material in spleen tissue of patient 2, on the thin-layer plate, detected carbohydrate-positive material at the start line suggestive of an accumulation of a carbohydrate-containing substance of high molecular weight. In contrast, the spleen tissue of two control individuals showed only small traces and thus distinctly less amounts of carbohydrate-positive material compared to the patient.

### Visceropathological investigations

**Fig. 4:**
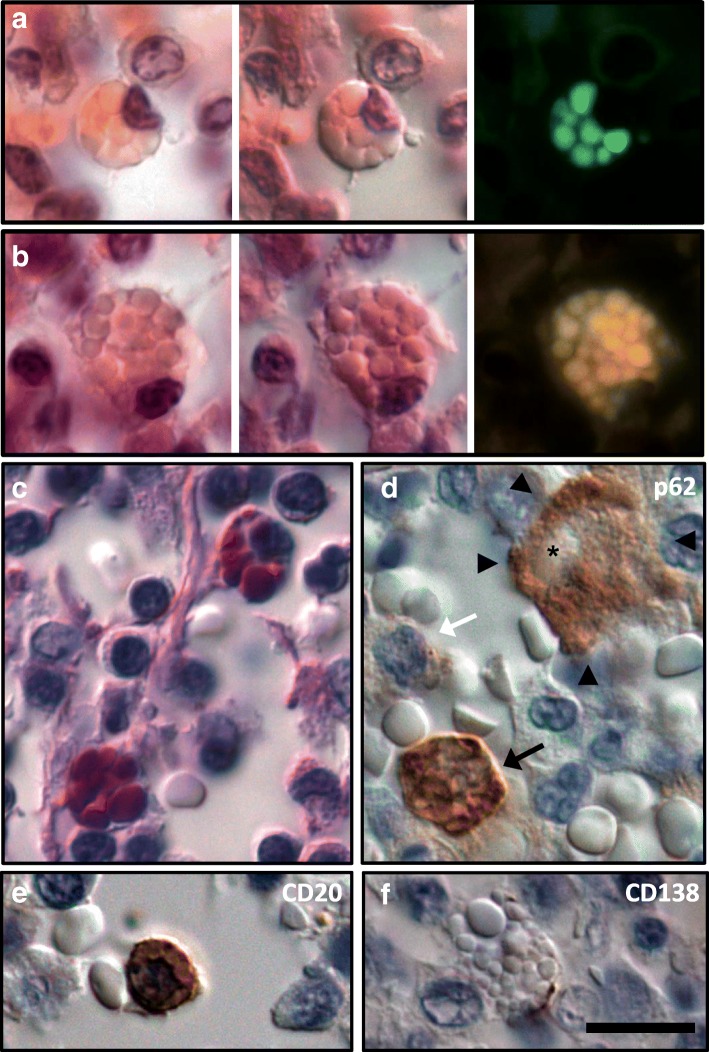
Vacuolated lymphocytes in lymph nodes of patient 1. A few lymphocytes with clear bold cytoplasmic vacuoles can be detected in the hematoxylin eosin staining in normal microscopy (**a** and **b**, first panel) and differential interference contrast (DIC) microscopy allowing for a three-dimensional illustration (**a** and **b**, second panel). The vacuoles exhibit a clear autofluorescence in different channels (**a** and **b**, right panel) and are strongly PAS-positive (**c**, DIC). An enhanced autophagosome formation is indicated by strong p62 immunoreactivity (**d**, black arrow), whereas normal lymphocytes are predominantly negative (white arrow). Note the positively stained larger macrophage (arrowheads) phagocytosing an erythrocyte (asterisk). Most of the vacuolated cells are CD20^+^ B-lymphocytes (**e**, DIC), and single cells show a partial CD138 expression, likely pointing at immature plasma cells (**f**, DIC). Scale bar: 10 μm

General autopsy of both patients revealed respiratory failure as cause of death, which was evident with multiple atelectases, recurrent small embolisms, pulmonary edema and single foci of pneumonia in patient 1 and with a fulminant bronchopneumonia in patient 2. Strikingly, in the obtained mesenterial lymph nodes few leucocytes contained large uniform bold vacuoles, which were pale in hematoxylin eosin staining, strongly PAS-positive and did not stain for chloracetate esterase (Fig. 4). They emitted a strong yellowish, green and red autofluorescence in light excitation with respective wave lengths. Immunohistochemical analysis characterized the vacuolated cells mainly as B-lymphocytes and as occasional plasma cells with an incomplete differentiation (Fig. 4e-f), whereas T-cells and macrophages were not affected. The vacuolated lymphocytes suggested an enhanced autophagosomal load, as indicated by p62 immunohistochemistry (Fig. 4d).

In both patients, the spleen showed subacute congestion. Here, single extracellular clusters of autofluorescent polymorphic structures suggestive of degraded material were seen (data not shown). The liver showed fatty degeneration, presenting as diffuse small lipid droplets in patient 1 and as centrolubular hypoxic fatty changes in patient 2. Neither in the spleen nor in the liver storage cells could be detected, but patient 2 exhibited PAS-positive material subendothelial to small arterioles in both organs. No lipopigments were seen in all investigated peripheral organs, including heart, lung, liver, spleen, kidneys, endocrine organs, gastrointestinal and urogenital tracts as well as skeletal musculature.

As individual pathologic changes, patient 1 showed a jejunal invagination with beginning impaired circulation and a reactive hyperplasia of single mediastinal, mesenterial and inguinal lymph nodes. Patient 2 had kidneys reduced in weight to 50% of the age related norm and a thoracolumbal scoliosis.

### Neuropathological investigations

**Fig. 5:**
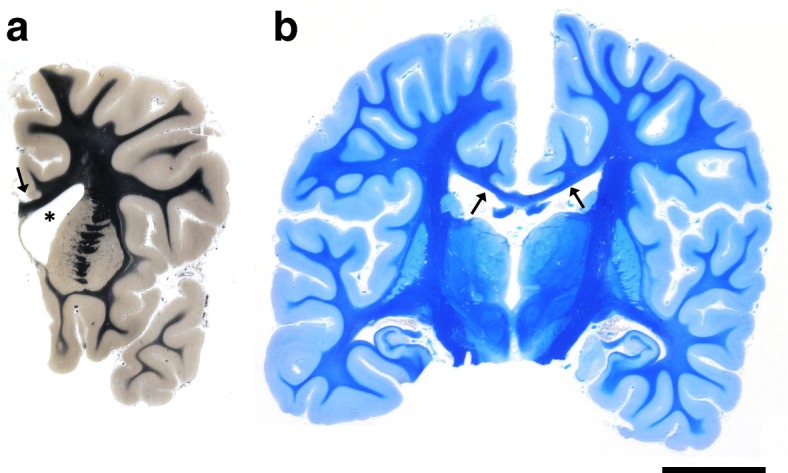
Whole mount coronar brain sections of patient 1 (**a**, Heidenhain-Woelcke stain) and patient 2 (**b**, luxol fast blue stain). The lateral ventricle is widened especially in patient 1 (**a**, asterisk). The brain of patient 2 shows narrowing of the white matter (**b**, blue staining of myelin). The corpus callosum is thinned in both patients (**a** and **b**, arrows). Scale bar: 2 cm

On autopsy, both brains showed a moderate global atrophy including a hydrocephalus internus and narrowed cortical gyri with dilated sulci in patient 1 and narrowing of white matter tracts in patient 2 (Fig. 5). Brain weights were appropriate to the age related norm (1490 g and 1350 g formalin fixed, respectively). The corpus callosum was thinned. Basal ganglia, thalami, hippocampi, brain stem and cerebellum appeared macroscopically unremarkable. The dorsal nerve roots of the spinal cord were partly thickened in patient 2. There was acute congestion with dilated capillaries. Singular microbleeds were found in the white matter of cerebrum, cerebellum and pons. In patient 1, individual hippocampal neurons of Sommer sector CA1 and of the nucleus dentatus were shrunken and hyperchromatic, indicating previous hypoxia. Patient 2 showed mild edematous changes in the cerebrum, a severe reduction of Purkinje cells with strong activation of Bergmann glia and a discrete fibrosis of leptomeninges. The white matter showed normal myelination.

Both patients showed a significant muscular atrophy, especially of the lower legs. Muscle specimens revealed a neurogenic atrophy with angular fibers and partial fiber groupings (data not shown).

Histological examination of the central nervous system (CNS) revealed one major pathological finding in both patients: cytoplasmic accumulation of granular storage material within neurons and to a lesser extent within glial cells. Interestingly, the number of neurons was normal to slightly reduced. The intracellular inclusions were distributed bihemispherically in many neurons of nearly all investigated brain regions. A detailed list is provided in Table 2. Most severely affected were the cerebral cortex laminae V and VI, claustrum, corpus geniculatum laterale, olivary nuclei, nuclei nervi vagi and the anterior horn of the spinal cord. The mesencephalon and the lateral and posterior horns of spinal cord were mainly preserved, the Purkinje cells peculiarly spared. The occurrence of neurons with abnormal storage material was more pronounced in patient 1 as compared to patient 2, who showed significantly less affected neurons in the cortical laminae I-IV, striatum and hippocampus.

**Table 2:** Distribution of neuronal storage material and PAS-positive granules in the central nervous system. Screening for neuronal inclusions was performed on sections stained with cresyl violet luxol fast blue (LFB) and sudan black (SB) or under ultraviolet light excitation for emission of autofluorescence (AF)

Region		Neuronal storage material		PAS + granules
		LFB/SB	AF	
Cerebrum				
Basal ganglia	Neocortex I – IV	–	++	++
	Neocortex V - VI	+++	+++	++
	Nucleus caudatus	(+)	+	–
	Putamen	–	++	(+)
	Claustrum	+++	+++	–
	Thalamus	++	+++	–
Hippocampus	CA 1	++	+++	+
	CA 2–4	+	++	+
	Corpus geniculatum laterale	+++	+++	n. a.
Cerebellum	Cortex			
	Purkinje cells	–	–	–
	Granule cells	–	–	–
	Subcortical white matter	–	–	+++
	Nucleus dentatus	(+)	++	+++
Brain stem				
Mesencephalon	Substantia nigra	–	(+)	++
	Nucleus oculomotorius	–	(+)	n. a.
Pons	Pontine nuclei	(+)	+	+++
	Raphe nuclei	(+)	+	+++
Medulla oblongata	Olivary nuclei	+++	+++	(+)
	Nucleus nervus vagus	+++	+++	–
	Nucleus nervus hypoglossus	–	++	–

Note the distinctly higher sensitivity of detection with autofluorescence. n. a., no available PAS-stained section or paraffin-embedded tissue

**Fig. 6:**
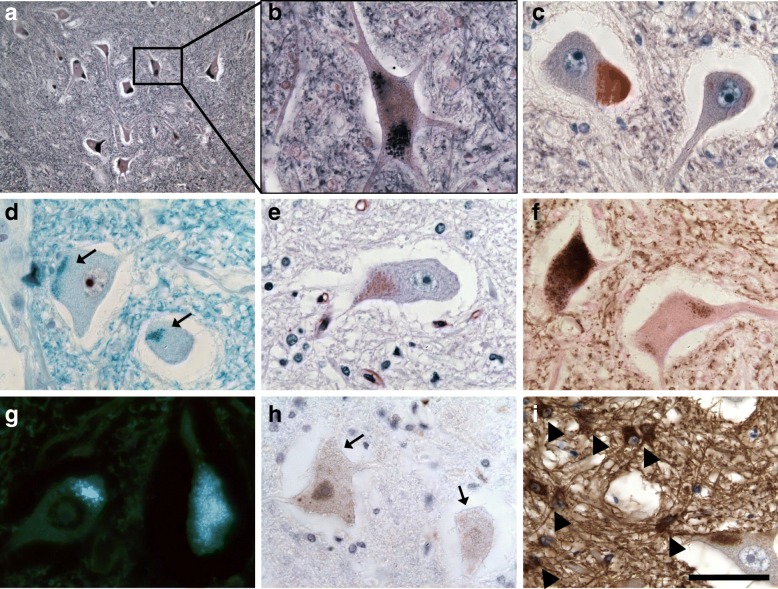
Neuronal inclusions in the spinal cord anterior horn of patient 2. Many neurons present with granular deposits in the perikaryon, which stain strongly with sudan black (**a** and **b**) and sudan red (**c**). Note the frequent localization adjacent to neuronal processes. The inclusions stain with luxol fast blue (**d**, arrows) and show a weak PAS-reaction (**e**). The storage material is moderately argyrophilic (**f**, Gallyas stain) and shows strong autofluorescence (**g**, unstained section). The storage material does not stain for p62 (**h**, arrows). Note the reactive GFAP^+^ gliosis (**i**, arrowheads). GFAP, glial fibrillary acidic protein. Scale bar: 250 μm in **a**, 50 μm in **b**-**i**

**Fig. 7:**
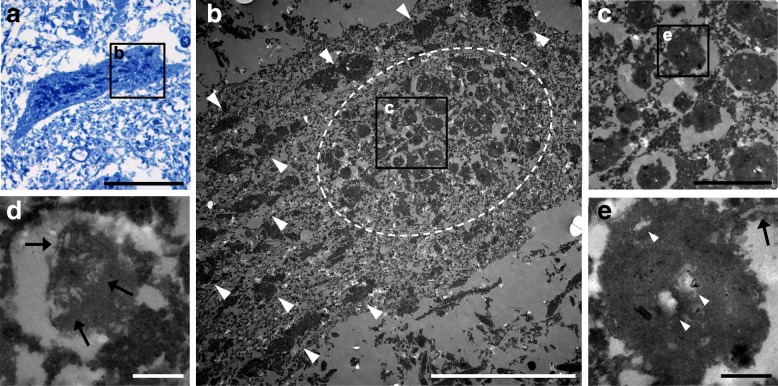
Ultrastructural morphology of neuronal storage material. Semi-thin section stained with toluidin blue of a neuron in the spinal cord anterior horn of patient 2 shows strongly stained storage material (**a**). Ultrastructural examination of the same neuron in a serial section (**b**-**e**) reveals a cluster of intracytoplasmic globular inclusions (delineated in **b**) consisting of amorphous osmiophilic material with high-density particles, lipid droplets (**e**; white arrowheads) and structures reminding of degraded membranous material (**d** and **e**; arrows). These inclusions correspond to lipofuscin granules in lysosomal residual bodies and remind of granular osmiophilic deposits (GRODs). Note the different polygonal shape of physiological Nissl substance (**b**; white arrowheads). Scale bar: 50 μm in **a**, 10 μm in **b**, 2 μm in **c**, 250 nm in **d** and **e**

**Fig. 8:**
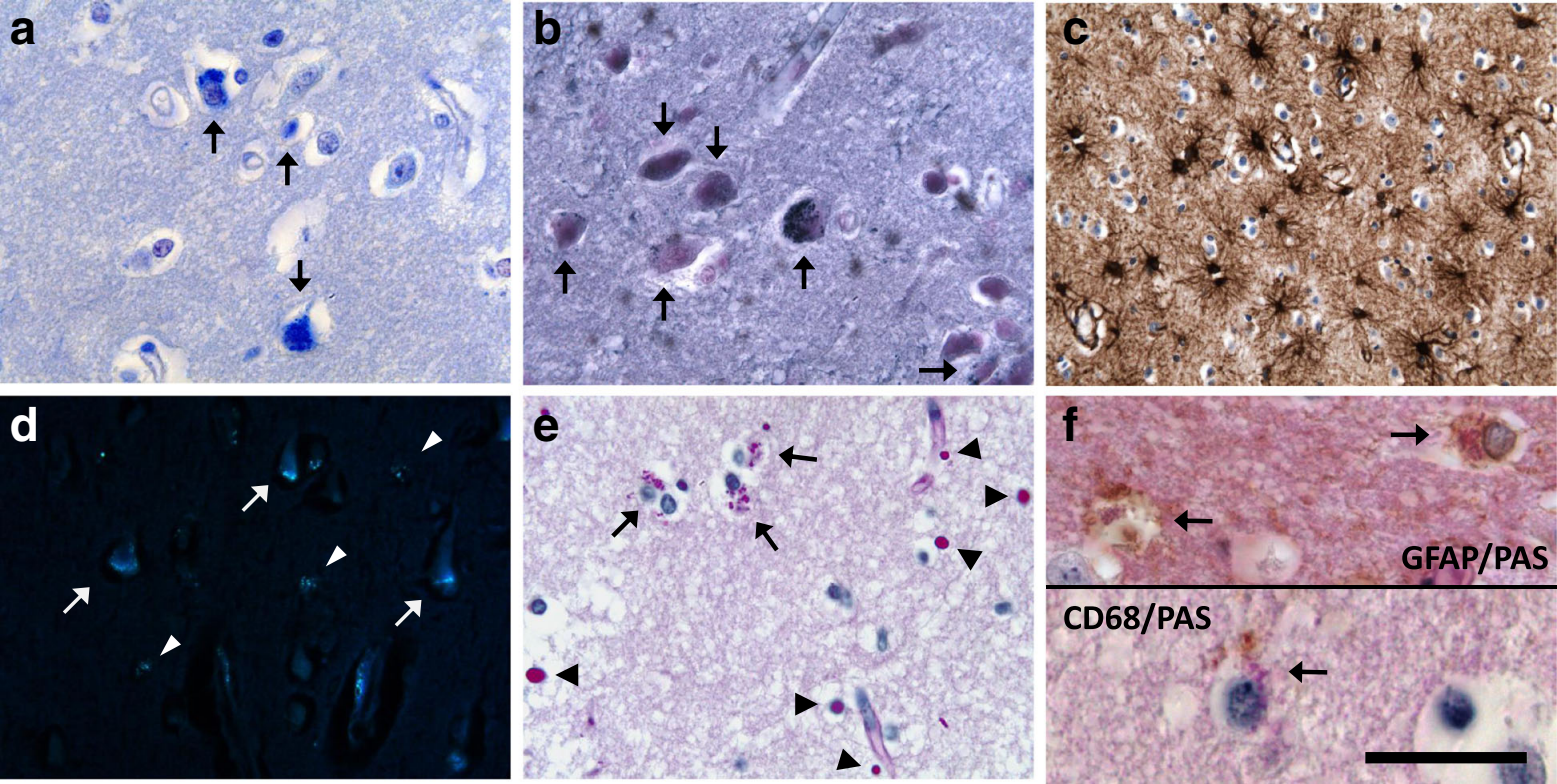
Morphological changes in the cortex of patient 2. Many cortical neurons in lamina V and VI incorporate storage material, which stains strongly with luxol fast blue in the Klüver Barrera staining (**a**, arrows) and with sudan black (**b**, arrows). A massive reactive astrocytosis is seen in GFAP immunohistochemistry (**c**). The intraneuronal inclusions (arrows) and glial inclusions (arrowheads) exert a strong autofluorescence (**d**, unstained section). Several cortical glia cells show cytoplasmatic PAS-positive granula (**e**, arrows). Note the numerous PAS-positive corpora amylacea (**e**, arrowheads). The laden glia cells are mainly astrocytes, as shown in double staining for PAS and GFAP (**f**, upper panel, arrows) and very few microglia cells, as shown in double staining for PAS and CD68 (**f**, lower panel, arrow). GFAP, glial fibrillary acidic protein. Scale bar: 50 μm in **a**-**e**, 20 μm in **f**

The neuronal inclusions were densely packed in the perikaryon and lay frequently adjacent to neuronal processes (Fig. 6). They had a round shape with an average diameter of 1 μm, varying from 0.3 to 2 μm. They stained strongly with luxol fast blue and sudan black and were argyrophilic. In unstained sections, they exhibited a bright silvery autofluorescence in ultraviolet light excitation and a weaker green and red autofluorescence in light excitation using respective lasers (Figs. 6 and 8 patient 1; Additional file 1: Supplement A patient 2). The detection of autofluorescent inclusions was most sensitive compared to other stains (Table 2). The PAS-reaction of the granules was heterogeneous and mainly weak (Fig. 6e), the alcian blue staining was entirely negative. A small proportion of the storage material was weakly immunoreactive for ß-amyloid, whereas a negative immunoreactivity was observed for ubiquitin, TDP-43 and the marker for autophagosomes p62 (Fig. 6h). In ultrastructural analysis, the inclusions presented as globular structures with a partially undulating border and a surrounding empty halo (Fig. 7). They consisted of amorphous osmiophilic, compact or granular material including high-density particles, lipid droplets (Fig. 7d-e) and sometimes membrane packages (Fig. 7d lower arrow), thus being highly suggestive of lipofuscin granules in lysosomal residual bodies. They furthermore showed a strong similarity to granular osmiophilic deposits (GRODs). Some neurons in addition contained vacuoles with membraneous structures resembling membranous cytoplasmic bodies (MCB) and zebra bodies (Additional file 1: Supplement B).

Intracellular storage material was not only present in neurons of both patients, but also in some glial cells. These inclusions varied in their features from those of neuronal storage material, as they showed a strong PAS reaction, no staining with luxol fast blue or sudan black/red and a silvery autofluorescence in ultraviolet light excitation of unstained sections (Fig. 8d-e). Double staining with PAS + GFAP and PAS + CD68 revealed a localization of the material in the cytoplasm mostly of astrocytes and to a lesser extent of microglia (Fig. 8f).

**Fig. 9:**
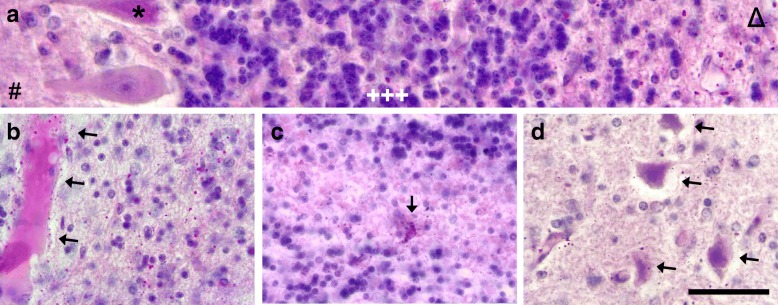
Diffuse PAS-positive material in the cerebellum of patient 1. The PAS-positive deposits are abundantly present in the subcortical cerebellar white matter (**a**, Δ; **b** and **c**) with sparing of molecular layer (#), Purkinje cell layer (*) and granular cell layer (+++). The deposits often accumulate perivascular (**b**, arrows) and are sometimes seen in glia cells (**c**, arrow). The nucleus dentatus is also affected (**d**). Note the shrunken and hyperchromatic neurons (arrows) as a sign of previous hypoxia. Scale bar: 65 μm in **a**, 50 μm in **b**-**d**

Finally, diffusely dispersed small granules of PAS- and Shimizu-positive material were observed in the neuropil of some areas in patient 1 and less frequent in patient 2. Affected regions were only partially concordant with the described areas showing neuronal inclusions, as the cerebellum and pons harbored the highest content of grains (Tab. 2, Fig. 9). The material lay diffusely in the grey and white matter of the mentioned regions with a notable dense perivascular accumulation (Fig. 9b). The remaining CNS showed isolated subendothelial aggregations. The deposits varied in size between 0.2 μm and 2 μm in diameter, the larger ones emitted autofluorescence. The granules were mainly globular, but large grains were often polygonal, allowing for discrimination against round corpora amylacea with a larger diameter. Considering the age of the patients, an excessive number of corpora amylacea was found in the brain and spinal cord of both patients, predominantly located in subpial, subependymal and perivascular areas.

An extensive reactive gliosis was present especially in the regions containing storage materials (Figs. 6i, 8c). Immunohistochemistry for CD68 revealed a moderate activation of microglia, other inflammatory changes were not seen.

## Discussion

This study provides a description of the morphological and biochemical pathology in the CNS and peripheral organs of patients with inherited TBCK-DD. The presence of 1) predominant lipofuscin-like storage material in CNS neurons, 2) storage deposits in astrocytes and to a lesser extent in microglia, 3) grainy PAS-positive deposits mostly in the pontine and cerebellar neuropil, spleen and liver, and 4) vacuolated lymphocytes support the classification of TBCK-DD as an LSD and give important evidence for the understanding of its pathogenesis.

### Morphological classification of TCBK-DD

#### Neuronal inclusions correspond to lipofuscin granules

The foremost observation were the frequent intraneuronal granular deposits spread throughout almost the whole central nervous system. Neuronal storage material is characteristic for LSDs, which comprise more than 50 diseases with a wide variation of storage products depending on defects in lysosomal enzymes, lysosomal membrane-associated proteins or non-lysosomal associated enzymes [33]. Here, positive staining for Sudan black and luxol fast blue, a moderate PAS reaction together with the absence of eosinophilia demonstrated that the inclusions consist predominantly of lipopigments with minor or no protein content [49]. In addition, the inclusion material showed strong autofluorescence, a characteristic feature of lipofuscin, the storage material in neuronal ceroid lipofuscinosis (NCL) and mucopolysaccharidosis type III (MPS III, Sanfilippo syndrome), respectively [12, 46, 52].

In concordance with the light microscopical characteristics, ultrastructurally the majority of storage material corresponded to lipofuscin granules in lysosomal residual bodies and in particular resembled granular osmiophilic deposits (GRODs) as seen in NCL types 1, 4, 5, 8–10, 12 and 14 [34]. Moreover, few zebra body- and MCB-like structures similar to inclusions in gangliosidoses and MPS were observed [16, 21]. In contrast to MPS, the neuronal deposits did not stain with Alcian blue and they showed strong autofluorescence distinguishing the disorder from gangliosidoses and all but one MPS (MPS III). Since the electron microscopical investigation was performed on material that was recovered from formalin fixed and paraffin embedded tissue, ultrastructural morphology was suboptimally preserved and therefore we cannot fully exclude the possibility that TBCK-DD pathology consists of further alterations.

The autofluorescent glial inclusions differed from the neuronal deposits in that they were PAS-positive and negative in luxol fast blue and Sudan black stains, suggesting that they contain a larger fraction of carbohydrates in addition to autofluorescent lipofuscin. The inclusions were mostly present in astrocytes, but also seen in few microglia.

#### Diffuse accumulation of PAS-positive material in the CNS and in peripheral organs

Moreover, PAS-positive granular deposits were observed diffusely in the neuropil. Interestingly, the spleen and liver contained PAS-positive granules in subendothelial areas, too. These deposits biochemically corresponded to increased concentration of carbohydrate-positive material in the spleen of patient 2 and indicate an insufficient degradation of saccharides or glycosylated substrates. Similarly, a recent study described enhanced oligosaccharide levels in fibroblasts and urine of patients with TBCK-DD [35]. The absence of urinary oligosaccharides in our patient 2 might be due to the lower sensitivity of the test used in 1987 as compared to the mass spectrometric analysis recently performed by Ortiz-Gonzalez et al. [35]. The distribution pattern of PAS-positive material was different in the two patients, indicating that these deposits might be a variable pathologic feature of TBCK-DD in different organs. The more severe brain affection of patient 1 correlated with the worse clinical course compared to that of her sister. All in all, storage of PAS-positive material was much less pronounced as in mucopoly−/oligosaccharidosis, gangliosidoses or polyglucosan body disease.

#### Vacuolated lymphocytes

Here, we discovered the presence of vacuolated B-cells and immature plasma cells in TBCK-DD. Vacuolated lymphocytes are a feature of several metabolic diseases including CLN3 [2, 3, 10, 27, 45] and CLN11 [8], Pompe’s disease/adult acid maltase deficiency [2, 19, 36], GM1 gangliosidosis [2, 14, 15] and others as summarized by Anderson et al. [2].

The autofluorescence together with the strong PAS-reaction of the vacuoles in lymphocytes of our patients suggest an accumulation of both lipopigment and carbohydrates. PAS-positive vacuoles in lymphocytes have been reported in patients with Pompe’s disease/adult acid maltase deficiency [2, 19] and Danon disease (LAMP2-deficient cardiomyopathy) [32]. At present it is unknown whether vacuolated lymphocytes are regularly present in the peripheral blood of patients with homozygous *TBCK* mutation. Should this be the case, peripheral blood film examination might represent a useful and simple diagnostic tool to support the diagnosis of TBCK-DD.

The vacuolated lymphocytes showed excessive p62-positive autophagosomes which have been previously described in TBCK-DD patients’ fibroblasts [35]. This contrasted to neurons that showed no accumulation of p62 immunoreactive organelles. This variance may be explained either by cell type-specific effects of TBCK or by alternative pathways regulating autophagy in postmitotic neuronal cells as compared to dividing cells such as fibroblasts [11, 28].

#### Synopsis of morphological changes

In summary, our findings revealed characteristic morphological changes in both patients with TBCK-DD that are typical for an LSD. Subclassification into a specific group, however, is not straightforward since we observed aggregates with different morphological properties pointing at different storage materials. While the majority of neuronal inclusions in the CNS indicate an NCL, the PAS-positive vacuoles in lymphocytes are reminiscent of those seen in Pompe’s disease and the PAS-positive aggregates in the CNS, spleen and liver as well as the occasional intraneuronal zebra bodies and MCB-like structures demonstrate carbohydrate-containing material as is stored in polyglycosan body diseases, mucopoly−/oligosaccharidosis or gangliosidosis. Together, TBCK-DD represents a new type of storage disorder, characterized by the occurrence of different storage products with predominance of lipofuscin. Future studies will show whether a classification as a novel subtype of NCL (CLN15) is appropriate.

### Metabolic consequences of TBCK defects

A recent study demonstrated an autophagosomal-lysosomal dysfunction in TBCK-DD with accumulation of autophagosomes and impaired degradation of glycosylated proteins in cultured fibroblasts [35]. Mechanistically, TBCK can be linked to autophagy as a regulator of two distinct pathways including mTOR and small GTPases of the Rab-family. Knockdown of TBCK in vitro significantly downregulated the main inhibitor for autophagy initiation, mTORC1 [4, 6, 29], thus explaining an enhanced production of autophagosomes in case of defective TBCK. Importantly, disturbed mTORC1 signaling and lysosomal dysfunction have been observed in a mouse model of CLN11/progranulin deficiency, underpinning the concept of TBCK-DD being an NCL [40, 43, 48]. Furthermore, TBCK has been proposed a putative activator protein for small GTPases of the Rab-family [9], which regulate fusion of autophagosomes with lysosomes [18, 22, 24, 47]. In line with this interpretation, Rab-associated dysfunctional endocytic membrane trafficking was described in *CLN*3 mammalian cells [30]. It is therefore likely that a TBCK defect leads to both, enhanced autophagosome formation and decreased fusion with lysosomes which in turn cause a disturbed clearance of cell components e.g. glycosylated substrates and an accumulation of non-degradable products such as lipofuscin.

In addition to the effects on autophagy, mTOR has been reported to mediate key endogenous neuroprotective mechanisms in motoneurons [41] and to contribute to peripheral axonal myelination and growth [44], thus possibly explaining the severe affection of the second motoneuron due to both CNS (storage inclusions) and peripheral (myelination and axonal growth) pathologies in TBCK-DD.

To date, few neurodevelopmental disorders have been linked to an aberrantly reduced mTOR signaling as seen in the herein described TBCK-DD, including Rett syndrome [38, 39], Phelan-McDermid syndrome with autism spectrum disorder [5] and Galloway-Mowat syndrome [23]. These diseases share the symptoms of cognitive deficits and epilepsy, but are not associated with an accumulation of storage products. Thus, the appearance of TBCK-DD as an LSD is likely to result from multifactorial TBCK-specific alterations, which need to be elucidated in further studies. So far, therapeutic mTORC1 activation may be a potential strategy to prevent disease progress in patients.

### Correlation of morphological changes to clinical symptoms

The presence of storage material in a large number of neurons in the absence of significant neuronal loss suggests neuronal dysfunction as the underlying cause of TBCK-DD. This is a clear difference to most other LSDs that are characterized by severe neuronal degeneration with marked brain atrophy in late stage disease. The distribution pattern of neuronal storage material fits well with the clinical phenotype: neurogenic atrophy of skeletal muscle is likely the consequence of secondary motoneurons in the spinal cord being severely affected and intellectual disability is consistent with the high amount of neuronal inclusions in the neocortex, archicortex and hippocampus. Those changes may not only explain neuronal deficits, but also account for uncontrolled neuronal excitations as a source of epileptic seizures. As the patients suffered from declining visual acuity or blindness, an involvement of the retina like in CLN1–3 and CLN5 [37] seems possible, although an electroretinogram in one patient with TBCK-DD was normal [9] and points to a cause by affected neurons in the central visual tract. Overall, the clinical symptoms with severe developmental delay and intellectual disability, hypotonia, severe visual deterioration and generalized seizures resemble those seen in infantile and late infantile CLN1 and CLN2, respectively.

## Conclusion

In conclusion, our investigations uncover TBCK-DD as a novel LSD. The predominant neuronal lipofuscin inclusions as well as the clinical symptoms are typical for an NCL and may indicate a novel subtype (CLN15). The accumulation of carbohydrate-related material and the PAS-positivity of lymphocytic vacuoles, however, exceed the pathological alterations seen in other NCL. Since our investigations are limited due to restrictedly archived tissue material, further studies, in particular ultrastructural analyses of the CNS and vacuolated lymphocytes are needed in order to come to a definite classification of this disorder. The underlying mechanism can be assigned to an autophagosomal-lysosomal dysfunction, including enhanced mTORC1-mediated autophagosome formation and reduced Rab-mediated autophagosome-lysosome fusion, thus implicating new targets for therapeutic approaches in TBCK-DD.

## Additional file

Additional file 1: Additional histological and ultrastructural observations. (PDF 771 kb)

## Abbreviations

CHp: Carbohydrate positive
CNS: Central nervous system
GROD: Granular osmiophilic deposit
IHPRF3: Infantile muscular hypotonia with psychomotor retardation and characteristic facies
LSD: Lysosomal storage disease
MCB: Membranous cytoplasmic body
MPS: Mucopolysaccharidosis
mTOR: mammalian target of rapamycin
mTORC1: mTOR complex 1
NCL: Neuronal ceroid lipofuscinosis
PAS: Periodic acid-Schiff
TBC: Tre-2/Bub2/Cdc16
TBCK: TBC1 domain-containing kinase
TBCK-DD: TBCK deficiency disorder
CLN1–15: type 1–15 of neuronal ceroid lipofuscinosis
