# Supplementary material for: Homozygous TBC1 domain-containing kinase (TBCK) mutation causes a novel lysosomal storage disease – a new type of neuronal ceroid lipofuscinosis (CLN15)?
Source: Acta Neuropathol Commun. 2018 Dec 27;6:145. doi: 10.1186/s40478-018-0646-6 (PMC6307319; doi:10.1186/s40478-018-0646-6)
Supplement: Supplementary file 1 — Additional histological and ultrastructural observations. (PDF 771 kb) [file 40478_2018_646_MOESM1_ESM.pdf]

Supplement A

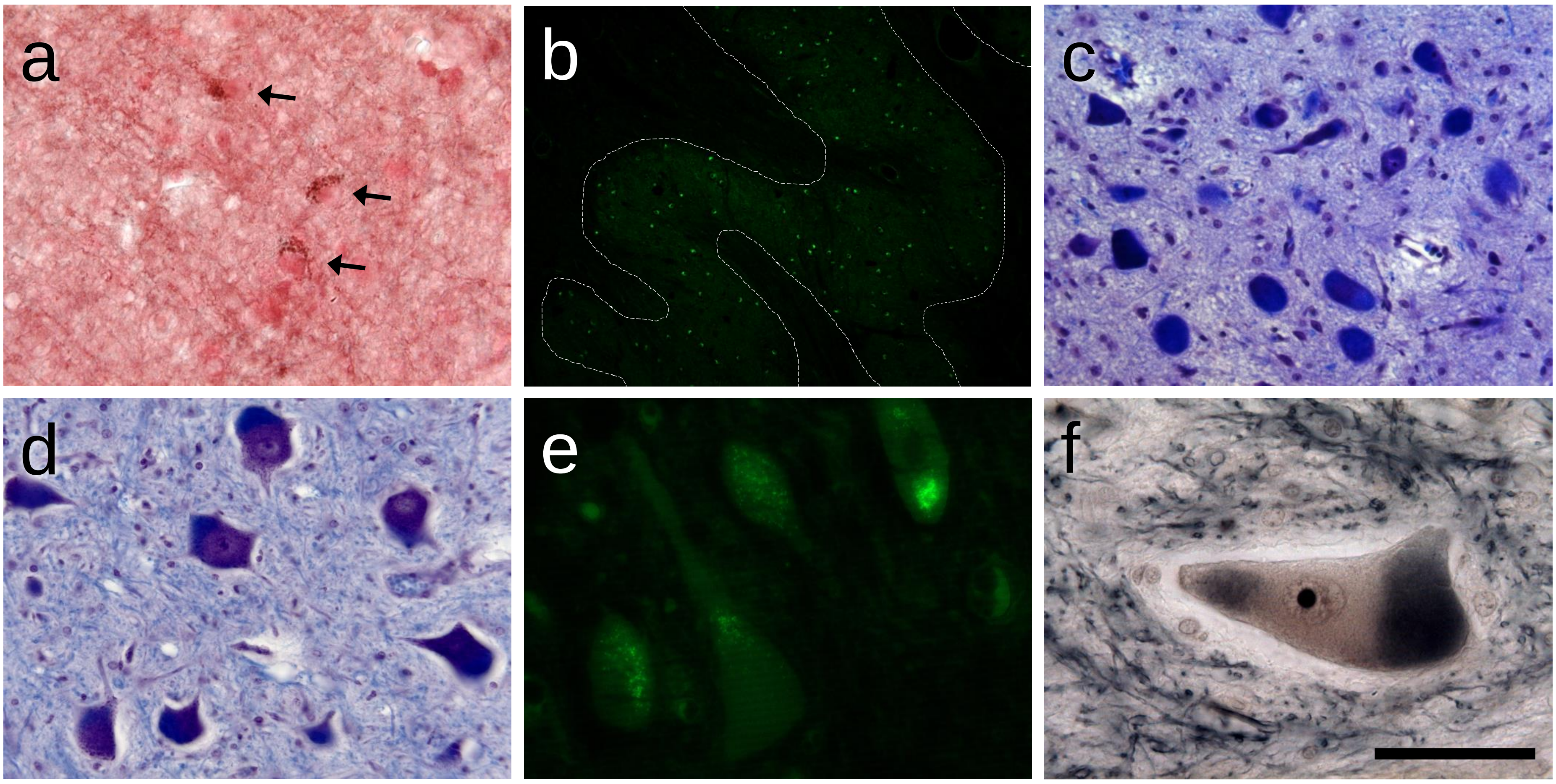

Neuronal storage material in patient 1. Neuronal inclusions with the same characteristics as those seen in her sister are present in the central nervous system of patient 1. The neurons were affected amongst other regions in the cortex (a, sudan black, arrows), nuclei olivary (b, green autofluorescence), nuclei nervi vagi (c, Kluver Barrera) and in the anterior horn of the spinal cord (d, Kluver Barrera; e, green autofluorescence; f, Heidenhain-Woelcke). Scale bar: 50 µm in a, e, f; 1000 µm in b; 125 µm in c, d.

Supplement B

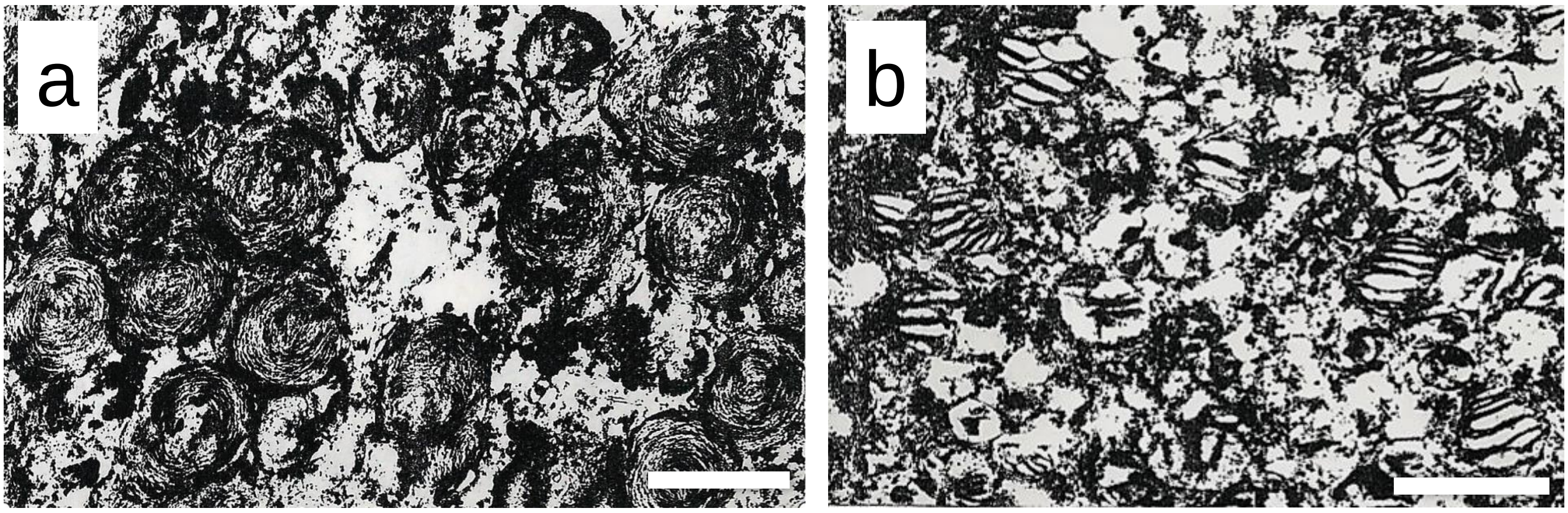

Ultrastructural morphology of neuronal storage material. Both panels show cytoplasmic inclusions in neurons of the spinal cord anterior horn in glutaraldehyde-fixed, resin-embedded material of patient 1. In addition to the abundant neuronal lipofuscin granules (*Fig. 6*), there were occasional structures resembling membranous cytoplasmic bodies (a) and zebra bodies (b). Scale bars: 1 µm.
